# Suicide in Brazilian indigenous communities: clustering of cases in children and adolescents by household

**DOI:** 10.11606/S1518-8787.2018052000541

**Published:** 2018-05-07

**Authors:** Thomas Adriano Lazzarini, Crhistinne Cavalheiro Maymone Gonçalves, Walter Martins Benites, Liliane Ferreira da Silva, Daniel Henrique Tsuha, Albert Icksang Ko, Robert Rohrbaugh, Jason Randolph Andrews, Julio Croda

**Affiliations:** I Yale University. School of Medicine. New Haven, Connecticut, USA; II Universidade Federal de Mato Grosso do Sul. Faculdade de Medicina. Campo Grande, MS, Brasil; III Distrito Sanitário Especial Indígena. Polo Base Dourados. Dourados, MS, Brasil; IV Universidade Federal de Mato Grosso do Sul. Faculdade de Computação. Campo Grande, MS, Brasil; V Fundação Oswaldo Cruz. Salvador, BA, Brasil; VI Division of Infectious Diseases and Geographic Medicine. Stanford University School of Medicine. Stanford, CA, USA; VII Fundação Oswaldo Cruz. Campo Grande, MS, Brasil

**Keywords:** Suicide, Child, Adolescent, Health of Indigenous Peoples, Mental Health, Health Vulnerability, Cohort Studies

## Abstract

**OBJECTIVE:**

To estimate age and sex-specific suicide rates, compare suicide rates between indigenous communities, and quantify the frequency of intrafamilial suicide clustering.

**METHODS:**

We performed a retrospective cohort study involving 14,666 indigenous individuals in reservations in Dourados, state of Mato Grosso do Sul, Brazil, from 2003 through 2013 using national and local census.

**RESULTS:**

The overall suicide rate was 73.4 per 100,000 person-years. Adolescent males aged 15-19 and girls aged 10-14 had the highest rates for each sex at 289.3 (95%CI 187.5-391.2) and 85.3 (95%CI 34.9-135.7), respectively. Comparing the largest reservations, Bororo had a higher suicide rate than Jaguapiru (RR = 4.83, 95%CI 2.85-8.16) and had significantly lower socioeconomic indicators including income and access to electricity. Nine of 19 suicides among children under 15 occurred in household clusters. Compared with adult suicides, a greater proportion of child (OR = 5.12, 95%CI 1.89-13.86, p = 0.001) and adolescent (OR = 3.48, 95%CI 1.29-9.44, p = 0.017) suicides occurred within household clusters.

**CONCLUSIONS:**

High rates of suicide occur among children and adolescents in these indigenous reservations, particularly in poor communities. Nearly half of child suicides occur within household clusters. These findings underscore the need for broad public health interventions and focused mental health interventions in households following a suicide.

## INTRODUCTION

Suicide is an important and preventable cause of mortality and is the second leading cause of death among those aged 15-29 worldwide^1^. High rates of suicide among indigenous adolescents is a public health problem documented in the United States, Canada, Australia, New Zealand, and Brazil^2^ ^-^ ^14^. In Brazil, where the overall suicide rate is relatively low (6.0 per 100,000 population per year)^1^, high rates of indigenous suicide have been reported in the Amazon and Midwest regions of the country^7^ ^,^ ^11^ ^-^ ^14^. In 2005, indigenous suicide rates in Mato Grosso do Sul (MS), a state in Midwest Brazil, were 10 times greater (86.3 per 100,000) than the overall rate in the state (8.6 per 100,000) and 19 times greater than the national rate (4.5 per 100,000 population in 2004)^14^.

While higher rates of suicide among indigenous communities compared to local non-indigenous populations have been well documented, there is limited understanding of which indigenous communities have the highest rates, and what differences exist between higher-risk and lower-risk communities, as this has received little attention. There is evidence of suicide clustering in indigenous communities^13^ and some indigenous families^15^ in the region, but the importance of intrafamilial clustering in child and adolescent suicide has not been explored.

To address these knowledge gaps, we investigated suicide patterns in five communities, two of which comprise the largest indigenous reservation in Brazil. Specifically, we aimed to estimate age and sex-specific suicide rates, compare suicide rates between communities of varying socioeconomic status, and quantify the amount of intrafamilial suicide clustering in the population.

## METHODS

The study was a retrospective cohort study, using mortality register data and population data to estimate age-specific suicide incidence rates among the indigenous populations served by the Dourados Indigenous Health Office (DSEI - *Polo Base Dourados*) between 2003 and 2013.

The study included five indigenous reservation communities, or *aldeias*, that comprise the service area for DSEI - *Polo Base Dourados* in MS, Brazil. Dourados is a municipality with a population of 210,000 in Midwest Brazil, near the Paraguay border. The vast majority of indigenous individuals in the region live on reservations, and the study focused only on indigenous individuals residing in reservation communities. The reservation population is ethnically heterogeneous with a Guarani-Kaiowá majority living alongside Terena and Guarani minorities. The Guarani-Kaiowá and Guarani groups have been in the region for centuries and most of the families living on the reservations were displaced from traditional lands within the last five decades. The Terena ethnic group is originally from northern MS and from the state of Mato Grosso.

Population and demographic data (age, gender, ethnicity, and address) were obtained electronically from the 2015 Special Indigenous Information System (SIASI) census and socioeconomic data (literacy rate, *per capita* income, and household data) were accessed electronically from the 2010 Brazilian National Census (IBGE). The SIASI population data were used because earlier data sets did not differentiate between indigenous ethnic groups. Unofficial population estimates and 2010 census figures suggest that the population has grown over the past two decades; therefore, using the 2015 population data to calculate rates likely resulted in lower reported rates. However, we considered this to be an acceptable trade-off for enabling the estimation of suicide rates stratified by ethnic group.

Suicide cases were drawn from The Brazilian National Mortality Database (SIM) and the SIASI suicide surveillance database. The SIM maintains demographic information (age, gender, marital status, ethnicity, and address) and ICD-10 codes for deaths in Brazil. Deaths with the suspicion of suicide undergo a forensic autopsy at the local Medical Legal Institute before being ascribed an ICD-10 code corresponding to “intentional self-harm” (X60-X84) in SIM. To avoid underreporting indigenous suicide, SIASI utilizes community health agents to investigate the circumstances of potential suicides with key informants. SIASI includes the individual's name, age, gender, ethnicity, suicide method, and address. Suicides occurring between 2003 and 2013 were extracted from each database. In the case of discrepancies, names, birth dates, and dates of death of the cases were compared to ensure that each was distinct.

The suicide rate in this population was calculated as suicides per 100,000 population per year. Suicide cases were extracted from SIM and SIASI, and population estimates were from the SIASI census. Comparisons of stratified suicide rates were assessed statistically using Pearson's chi-squared test. Statistical significance was defined as a relationship with p-value ≤ 0.05. Rate ratios and the 95% confidence interval for rate ratios were calculated according to the following equations^16^:

RR=abcd95%CI=exp(LN(RR)±1.96*1a+1b+1c+1d

Statistical significance was defined as rate ratios with 95% confidence intervals that did not include 1.00.

Household clustering of child and adolescent suicide was assessed by calculating the proportion of suicide victims who shared an address with another suicide victim during the study period. For this analysis, unique households were identified by the addresses listed for each suicide case in the SIASI database. The proportion of suicides that occurred in such a cluster was compared for different age strata, using Pearson's chi-squared test and expressed in odds ratios, to determine if household clustering of suicide specifically affected children and adolescents relative to other age groups.

This study was approved by the ethics committee at Universidade Federal de Mato Grosso do Sul, the Brazilian National Research Ethics Committee, the Brazilian Indigenous Research Ethics Committee (CAAE 35421914.7.0000.0021), and the Yale University Institutional Review Board. Neither the Bertram Roberts Memorial Fund nor the Wilbur Downs Fellowship had a role in designing or writing this report.

## RESULTS

In 2015, the indigenous population of the Dourados region consisted of 14,606 people living predominantly in five aldeias ranging in size from 49-1,280 households; a minority (824; 5.6%) lived outside these five *aldeias*. Guarani-Kaiowá were the majority ethnic group (88.1%-95.6% of the populace) in four of the five *aldeias*, and a minority (29.3%) only in Jaguapiru. Living conditions were poor in all *aldeias*; 47.9%-83.7% of the population had a *per capita* income of less than a quarter of the monthly minimum wage ($72.71 USD or R$127.50)^17^, 6.1%-52.3% have a private bathroom for the home, and 18.4%-89.7% have electricity in the home.

**Figure 1:**
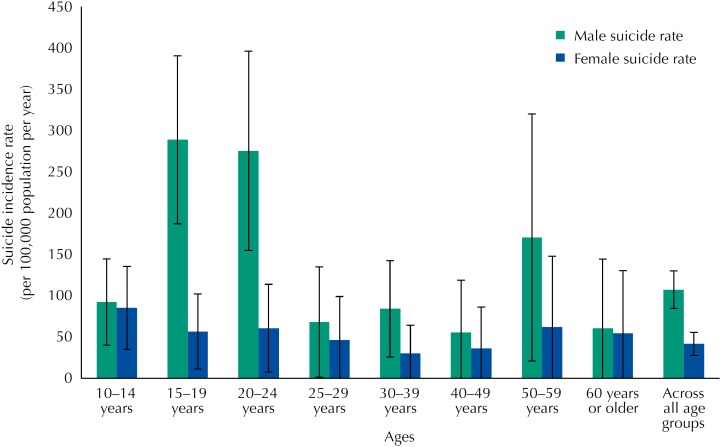
Crude suicide rate in indigenous reservations near Dourados, state of Mato Grosso do Sul, Brazil, according to age from 2003 to 2013.

A total of 119 suicide cases were identified in the *aldeias* between 2003 and 2013 for an overall suicide rate of 74.1 per 100,000 population per year. Most cases (93%; 111/119) were reported as hanging and the remaining 7% of cases (8/119) were reported as intentional agrochemical ingestion. The male suicide rate (107.5 per 100,000) exceeded twice the female rate (41.7 per 100,000); age group stratified male and female suicide rates are listed in Table 1 and depicted in Figure 1. The highest rates of suicide among males were observed for those aged 15-19 and 20-24 with rates of 289.3 per 100,000 (95%CI 187.5-391.2) and 275.9 per 100,000 (95%CI 155.0-396.8), respectively. These rates were significantly higher than all other male cohorts except ages 50-59. The highest rate among females was seen for children aged 10-14, whose rate was 85.3 (95%CI 34.9-135.7), which was not significantly greater than other female cohorts and was similar to the male suicide rate for the same age group (92.5 per 100,000). A higher proportion of female suicides, compared to male suicides, occurred among children under fifteen years of age (32.4% *versus* 14.1%, p = 0.044). More than half (50.4%; 60/119) of all suicides were among individuals under the age of 20.

**Table 1:** Crude suicide rate (2003-2013) and socioeconomic data (2010 census) for indigenous reservations near Dourados, state of Mato Grosso do Sul, Brazil.

*Aldeia*	Population^a^	Number of suicide cases^b^ (PY)^c^	Rate	95%CI	Relative to Jaguapiru		Distance from Dourados^e^	Guarani-Kaiowá percentage of population^a^	Male HH^f^	Percentage of households earning less than ¼ minimum wage *per capita* f	Have a private bathroom^f^	Have electricity^f^
					RR^d^	p						
Bororo	6,091	77 (67,001)	114.9	89.3-140.6	4.83	< 0.001	12 km	88.1%	53.1%	57.7%	31.6%	71.0%
Jaguapiru	6,495	17 (71,445)	23.8	12.5-35.1	Reference	–	5 km	29.3%	63.9%	47.9%	41.1%	89.7%
Panambi	547	10 (6,017)	166.2	63.2-269.2	6.97	< 0.001	39 km	91.8%	61.7%	52.5%	52.3%	80.5%
Panambizinho	334	8 (3,674)	217.7	66.9-368.6	9.13	< 0.001	19 km	95.6%	80.3%	49.4%	14.8%	65.4%
Sucuri	315	3 (3,674)	86.6	0.0-184.6	3.64	0.027	90 km	90.8%	97.9%	83.7%	6.1%	18.4%
Others	824	3 (9,064)	33.1	0.0-70.6	1.39	0.60	–	90.5%	–	–	–	–
Total	14,606	118 (160,666)	73.4	60.2-86.7	–	–	–	62.5%	–	–	–	–

PY: people-years; HH: head of household

a Population and ethnicity data from SIASI - FUNASA/MS 2015.

b Number of cases from SIASI and SIM databases (2003-2013) with reported *aldeia* of residence.

c People-years of follow-up calculated using population data from SIASI - FUNASA/MS 2015.

d Relative risk was calculated for Jaguapiru *versus* other *aldeias*.

e Distance from Dourados from the 2014 SESAI report.

f Percentage of households with a male head of household, percentage of population living on less than a quarter minimum wage, and other household data from 2010 IGBE national census for sectors that are 100% indigenous and located within the indicated reservation *aldeias*.

*Aldeia*-stratified suicide rates are shown in Table 2. Only one case was not linked to an address and was therefore excluded from the *aldeia*-stratified analysis. The highest suicide rate was seen in Panambizinho (218 per 100,000), followed by Panambi (166 per 100,000), Bororo (115 per 100,000), Sucuri (87 per 100,000) and Jaguapiru (24 per 100,000), indicating a nine-fold difference in suicide rate between the highest and lowest burden *aldeias*. The highest suicide rates were seen in the *aldeias* with the highest proportion of Guarani-Kaiowá, and the lowest suicide rate was observed in Jaguapiru, which is the only *aldeia* in which Guarani-Kaiowá are a minority. It also has the highest proportion of households with electricity and the lowest rates of extreme poverty. These *aldeia*-level trends in suicide rate were also seen when the Guarani-Kaiowá suicide rates were compared between the aldeias. The ethnicity-specific rates were all calculated to be lower than the overall suicide rates because no ethnicity was documented for 12.3% of the suicide cases. There were significantly higher rates of Guarani-Kaiowá suicide in Bororo (Rate = 105.0 per 100,000; RR = 5.50; p = 0.002), Panambi (Rate = 163.0 per 100,000; RR = 8.53; p < 0.001), and Panambizinho (Rate = 199.5 per 100,000; RR = 10.43; p < 0.001) than in Jaguapiru (Rate = 19.1 per 100,000). The Guarani-Kaiowá suicide rate was also higher in Sucuri (Rate = 63.6 per 100,000; RR = 3.33; p = 0.141) than in Jaguapiru but the result was not significant.

**Table 2:** Crude suicide rate (2003-2013) for the indigenous reservations near Dourados, state of Mato Grosso do Sul, stratified according to age and sex.

Age Group	Male			Female			Male *versus* Female		
	No. Cases^a^ (PY)^b^	Rate	95%CI	No. Cases^a^ (PY)^b^	Rate	95%CI	p^c^	RR^d^	95%CI^e^
All Suicides	85 (80,806)	107.5	84.7-130.4	34 (81,609)	41.7	27.7-55.7	< 0.0001	2.58	1.73-3.84
10-14 years	12 (12,969)	92.5	40.2-144.9	11 (12,892)	85.3	34.9-135.7	0.85	1.08	0.48-2.46
15-19 years	31 (10,714)	289.3	187.5-391.2	6 (10,560)	56.8	11.4-102.3	< 0.0001	5.08	2.12-12.17
20-24 years	20 (7,249)	275.9	155.0-396.8	5 (8,228)	60.8	7.5-114.0	0.0009	4.53	1.70-12.07
25-29 years	4 (5,863)	68.2	1.4-135.1	3 (6,446)	46.5	0.0-99.2	0.59	1.47	0.33-6.55
30-39 years	8 (9,625)	84.3	25.9-142.7	3 (9,933)	30.2	0.0-64.4	0.11	2.79	0.74-10.51
40-49 years	3 (5,357)	55.8	0.0-118.9	2 (5,522)	36.2	0.0-86.4	0.64	1.54	0.26-9.21
50-59 years	5 (2,959)	170.9	21.1-320.7	2 (3,223)	62.1	0.0-148.1	0.21	2.75	0.53-14.17
60+ years	2 (3,399)	60.6	0.0-144.6	2 (3,652)	54.8	0.0-130.7	0.92	1.11	0.16-7.85

PY: people-years

a Number of cases from SIASI and SIM databases (2003-2013) stratified according to age and gender.

b People-years of follow-up calculated using population data from SIASI - FUNASA/MS 2015.

c p-value calculated using Pearson's chi-squared test with statistical significance defined as p < 0.05.

d Rate ratio was calculated from the male and female suicide rates for the different age strata.

e 95% confidence interval of rate ratio between male and female suicide rates for different age strata.

**Figure 2:**
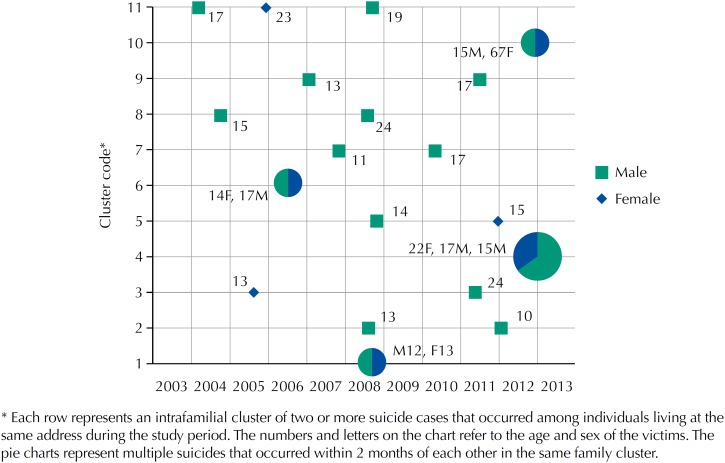
Household suicide clusters occurring in the indigenous reservations near Dourados, state of Mato Grosso do Sul, from 2003 through 2013. * Each row represents an intrafamilial cluster of two or more suicide cases that occurred among individuals living at the same address during the study period. The numbers and letters on the chart refer to the age and sex of the victims. The pie charts represent multiple suicides that occurred within 2 months of each other in the same family cluster.

Household clustering of suicides (Figure 2) was observed; 23.1% (24/104) of suicide victims had the same home address as another suicide victim during the study period. There was a total of eleven household clusters identified - nine with two cases each and two with three cases. The proportion of suicides occurring within such clusters was significantly greater among children under age 15 (47.4%; 9/19; p = 0.001) and adolescents between age 15 and 19 (32.3%; 10/31; p = 0.017) than among adults over the age of 20 (9.3%; 5/54). The mean time between the first and second suicide in the eleven household clusters was 830 days with a range of zero to 2102 days. Four of the secondary suicide cases took place within one month of the index household case (0, 1, 15, and 29 days apart, respectively).

## DISCUSSION

Three important observations in this study are the extremely high rate of suicide among children in this population, the frequent occurrence of household suicide clustering among children and adolescents, and the five-fold difference in suicide rates in neighboring communities with different socioeconomic conditions.

High rates of childhood suicide affect both sexes in the study population. Males aged 15-24 commit suicide at 36 times the national rate for their age group (7.9 per 100,000) and females aged 15-24 commit suicide at 25 times the national rate for their age group (2.3 per 100,000)^18^. During childhood (age 5-14), the discrepancy is even greater with both boys and girls in this population committing suicide at a rate more than 100 times higher than the national average for their age range (0.3 per 100,000 for both sexes)^18^. With a suicide rate of 85.3 per 100,000 people, girls aged 9-14 in this population commit suicide at a rate far greater than the suicide rate for indigenous girls in the entirety of Mato Grosso do Sul state (38.3 per 100,000)^13^. Female children in this population face many challenges, including high rates of sexual violence as well as childhood and teen pregnancy^19^. Further investigation is needed to understand the role that these stressors play in mediating suicide risk in female children in this population, as well as to identify additional potential suicide risk factors for girls.

We found dramatic evidence for household clustering of suicide cases. If suicides had occurred at random in households at the rates observed in this community, less than 0.3% would have clustered within the same household during the study period. By contrast, we found that 23.1% of all suicides occurred among individuals living at the same address as another suicide case. Approximately half of all child suicides (under age 15) and a third of adolescent suicides (age 15 to 19) occurred in familial clusters; whereas less than 10% of adult suicides (age 20 and above) occurred in such clusters. We were not able to assess the social relationships of all suicides in this study, but the role of suicide clustering in this population may, in fact, be much larger if social groups and extended families were to be considered. These data suggest that the experience of witnessing or grieving a suicide impacts an individual's subsequent risk of committing suicide in this population and that children and adolescents are particularly vulnerable.

Our findings corroborate evidence that poverty and socioeconomic disadvantage, as well as historical and cultural factors, increase an individual's risk of committing suicide (20-24). The neighboring *aldeias* of Bororo and Jaguapiru jointly comprise the Dourados Indigenous Reservation and represent 85% of the study population. The two *aldeias* share many commonalities in terms of size, location, a popularly held belief that indigenous culture has been “lost”, and prevalent alcoholism and drug abuse. However, the suicide rate in Bororo is nearly five times the rate in Jaguapiru. An obvious consideration is that demographic differences, namely the percentage of the population belonging to the Guarani-Kaiowá ethnic group, explain the difference in suicide rate. Bororo is almost 90% Guarani-Kaiowá while Jaguapiru is 30% Guarani-Kaiowá; however, the ethnicity-specific suicide rates calculated in this study do not support this hypothesis. A statistically significant five-fold difference in suicide rates between the two *aldeias* persists even when looking specifically at the Guarani-Kaiowá suicide rate.

The comparison between Bororo and Jaguapiru raises important questions regarding the interrelatedness of structural barriers like the dearth of educational opportunities and the challenges of accessing employment opportunities in the city, with indicators of poor well-being, such as the disintegration of families and suicide. Individuals living in Bororo experience higher structural barriers to economic success compared to their neighbors in Jaguapiru. Available data such as literacy rates and household income demonstrate that there are significant differences in the socioeconomic conditions and educational opportunities in the two *aldeias*. The implication is that youth without an education and without job prospects feel hopeless and that this hopelessness relates to the phenomenon of suicide.

## PUBLIC HEALTH IMPLICATIONS

When combined with the literature on indigenous suicide prevention program efficacy^9^ ^,^ ^25^ ^-^ ^30^, the findings of this study suggest potential pathways for addressing the indigenous child and adolescent suicide epidemic in Dourados that may also be applied more broadly to other indigenous communities with similar suicide epidemiology. Some specific interventions could be effective. First, given the young age at which suicides occur in the *aldeias*, a school-based intervention aimed at increasing knowledge of suicide and educating children and youth about local resources and how to disclose suicidal feelings to adult mentors and family members could be effective. Second, systematic screening has been effective in non-indigenous populations^30^ and could identify those at-risk of suicide as well as determine the prevalence of other behaviors such as substance abuse, self-harm, and suicidal ideation. Despite its prevalence, suicide may represent the tip of the iceberg of child psychosocial distress in this population. For this reason, community-wide screening would be tremendously informative. Large-scale screening may be prohibitively expensive, but the potential benefit is great if screening is targeted at children in Bororo. A final possibility, and one that has already been implemented, is timely intervention with friends and relatives of suicide victims. Currently, the *aldeias'* psychologist meets with those affected by a suicide to reconstruct the circumstances that preceded the suicide and assess proximate individuals’ need for counseling. It will be important to conduct a follow-up assessment of intrafamilial clustering to see if this intervention has been effective.

Two limitations may render the high suicide rates presented in this study as underestimations of the true burden of suicide in this population. Unreported or mischaracterized deaths may have been missed. We tried to maximize our case detection by using two separate databases, so it is likely that only a few cases were missed (estimated to be 2.9 by capture-recapture). Another limitation is that the 2015 population data were used in our calculations. These data were used because they are the earliest that delineate between specific ethnic groups, a variable that is crucial to understanding the population's suicide epidemiology. Unofficial population estimates and 2010 census figures suggest that the population has grown over the past two decades; therefore, utilizing 2015 population data to calculate rates resulted in lower reported rates.

By investigating *aldeia*-level and ethnic group differences in suicide epidemiology, this study was able to raise important questions regarding structural barriers that increase exposure of whole communities of children and youth to known suicide risk factors. Lack of access to education and employment results in decreased socioeconomic opportunities, poverty, and community and family disintegration. This may predispose children and adolescents to substance abuse, hopelessness, and ultimately suicide. Therefore, we strongly recommend interventions that reach these vulnerable young people and decrease the structural barriers to economic success, specifically improving access to educational and employment opportunities.
